# Vaccine value profile for Chikungunya

**DOI:** 10.1016/j.vaccine.2023.07.069

**Published:** 2024-07-25

**Authors:** Ximena Flandes, Clairissa A. Hansen, Sunil Palani, Kaja Abbas, Cate Bennett, William Perea Caro, Raymond Hutubessy, Kanat Khazhidinov, Philipp Lambach, Clara Maure, Caroline Marshall, Diana P. Rojas, Alexander Rosewell, Sushant Sahastrabuddhe, Marta Tufet, Annelies Wilder-Smith, David W.C. Beasley, Nigel Bourne, Alan D.T. Barrett

**Affiliations:** a Department of Preventative Medicine and Population Health and University of Texas Medical Branch, Galveston, TX, United States; b Department of Pathology, University of Texas Medical Branch, Galveston, TX, United States; c Department of Microbiology and Immunology, University of Texas Medical Branch, Galveston, TX, United States; d Faculty of Epidemiology and Population Health, London School of Hygiene and Tropical Medicine, London, United Kingdom; e School of Tropical Medicine and Global Health, Nagasaki University, Nagasaki, Japan; f Gavi the Vaccine Alliance, Geneva, Switzerland; g World Health Organization, Geneva, Switzerland; h International Vaccine Institute, Seoul, Republic of Korea; i Sealy Institute for Vaccine Sciences, University of Texas Medical Branch, Galveston, TX, United States; j Department of Pediatrics, University of Texas Medical Branch, Galveston, TX, United States

**Keywords:** Chikungunya, Vaccine, Virus infection, Vaccine value

## Abstract

Chikungunya virus (CHIKV) a mosquito-borne alphavirus is the causative agent of Chikungunya (CHIK), a disease with low mortality but high acute and chronic morbidity resulting in a high overall burden of disease. After the acute disease phase, chronic disease including persistent arthralgia is very common, and can cause fatigue and pain that is severe enough to limit normal activities. On average, around 40% of people infected with CHIKV will develop chronic arthritis, which may last for months or years. Recommendations for protection from CHIKV focus on infection control through preventing mosquito proliferation. There is currently no licensed antiviral drug or vaccine against CHIKV. Therefore, one of the most important public health impacts of vaccination would be to decrease burden of disease and economic losses in areas impacted by the virus, and prevent or reduce chronic morbidity associated with CHIK. This benefit would particularly be seen in Low and Middle Income Countries (LMIC) and socio-economically deprived areas, as they are more likely to have more infections and more severe outcomes.

This ‘Vaccine Value Profile’ (VVP) for CHIK is intended to provide a high-level, holistic assessment of the information and data that are currently available to inform the potential public health, economic and societal value of vaccines in the development pipeline and vaccine-like products. This VVP was developed by a working group of subject matter experts from academia, non-profit organizations, public private partnerships, and multi-lateral organizations. All contributors have extensive expertise on various elements of the CHIK VVP and collectively aimed to identify current research and knowledge gaps. The VVP was developed using only existing and publicly available information.

## 1 The global public health need for a vaccine

Chikungunya virus (CHIKV), the causative agent of the illness Chikungunya (CHIK) is transmitted to humans by virus-infected mosquitoes including *Ae. aegypti,* which can be found in tropical and sub-tropical regions, and *Ae. albopictus*, which are common in temperate regions, including several European countries and North America. The geographic ranges of both of these vector species are spreading, increasing the number of people who are at risk for infection [1], [2].

Although mortality due to CHIK is low, morbidity and the overall burden of disease is high. Symptoms of acute disease include high fever (above 39 °C), myalgia, arthralgia, arthritis, stiffness, headache, rash, insomnia, and exhaustion post-viremia [3], [4]. After the acute disease phase, chronic disease including persistent arthralgia and stiffness are very common. Arthralgia is likely to cause severe enough fatigue and pain to limit normal activities [5], and 87–98% of cases include joint pain [5]. On average, around 40% of people infected with CHIKV will develop chronic post-CHIK arthritis, which may last for months or years [3], [6], [7].

CHIK is a major public health problem. Between 2010 and 2019 it caused an estimated average annual loss of over 106,000 disability-adjusted life-years (DALYs) in the African, Americas, Eastern Mediterranean, European, Southeast Asian, and Western Pacific regions [6], with most of the losses resulting from long-term rheumatic sequelae after infection. However, there is potentially reporting bias towards reporting of long-term sequelae, which may overestimate DALYs. In addition to the burden of disease in endemic areas CHIKV has demonstrated the ability to produce large and sustained outbreaks/epidemics when introduced into new regions. For example, outbreaks in the Union of Comoros, La Reunion Island, and southern India during 2004–2009 resulted in over 2 million cases of CHIK. During such outbreaks/epidemics, the entire immune naïve population in the area is at risk of infection [8]. However, certain populations are at increased risk for more severe disease outcomes. These include individuals > 60 years (this population worldwide is expected to almost double from 12% to 22% by 2050) [9], [10], [11] and individuals who are overweight and obese (BMI > 25; >30 kg/m^2^) [9].

Despite the clear need, there are currently no licensed vaccines for CHIK. One of the most important public health impacts of vaccination would be to prevent or reduce CHIKV- associated chronic morbidity. This benefit would particularly be seen in low- and middle-income countries (LMICs) and socio-economically deprived areas, as they are more likely to have more infections and more severe outcomes [9].

### 1.1 Current methods of surveillance, diagnosis, prevention, and treatment

Surveillance for CHIK in various countries/regions generally relies on a combination of vector surveillance methods and active or passive surveillance for human cases. Diagnosis of CHIKV infection is typically based on criteria established by WHO and/or national health authorities that include: presence of relevant clinical manifestations (such as acute fever and arthralgia); epidemiological knowledge of possible transmission route, such as living in or visiting endemic areas; and subsequent laboratory diagnosis, which includes RT-PCR, IgM antibody detection, and/or virus isolation. A “probable” case would require clinical and epidemiological criteria to be present, while a “confirmed” CHIK case includes positive laboratory diagnosis. As CHIKV spreads, the disease has been classified as a “notifiable disease” in most countries.

Strategies to reduce CHIKV transmission and infection occur at an individual and community level. Because CHIKV is a mosquito-borne disease transmitted during daytime, it is recommended for individuals to engage in mosquito control interventions, such as removal of stagnant water, increase in gardening, use of safe aerosol sprays and mosquito repellent, installation of nets on household entry points, and increase in clothing covering [54]. In addition, community and government level interventions have focused on public sanitation and waste management improvement to remove breeding grounds for vectors.

As of July 1, 2023, there is neither licensed vaccine nor antiviral treatment for CHIK. Current treatment is supportive and includes paracetamol/acetaminophen for fever and pain, as well as light exercise and rest, which are recommended to start immediately when a case is suspected. NSAIDs are effective to treat arthralgia in CHIK patients, but these drugs should be avoided during the acute stage of disease and/or until official diagnosis is provided – due to possible confusion with dengue, which increases risk of hemorrhaging if steroids or similar drugs are utilized [14]. It is recommended to seek medical help if symptoms continue or worsen after five days, where NSAIDs regimen would be presented to patient under professional surveillance [55].

### 1.2 Summary of knowledge and research gaps in epidemiology, potential indirect public health impact and economic burden

• It is difficult to predict outbreaks of CHIK [3].
  o Outbreaks can be rapid and sporadic.
  o In naïve populations, outbreaks can affect 50–70% of the population.
  o Many areas have low ability to control outbreaks as the virus spreads from the sylvatic to urban transmission cycles.
• It is difficult to ascertain the level of endemic spread and annual average cases.
  o Most of the data come from estimated incidence and mortality rates during epidemics (see above). Also, much of the data are based on clinically suspected cases, which may not have been confirmed with laboratory-based methods [25], [26], [27].
  o There is limited information on CFRs for populations other than neonates and some elderly populations.
  o Limited availability of diagnostics and similarity of early clinical signs to other febrile infections leads to misdiagnosis of CHIKV infection and impacts collection of epidemiological data.
• There is little environmental surveillance for CHIKV, especially in lower income areas/regions [25], [26], [27].
  o It is difficult and resource intensive to test all possible animal reservoirs and maintain mosquito sampling for evidence of CHIKV infection/transmission year-round.
  o This also coincides with lack of vector control.
  o Surveillance systems are often clinic or hospital-based, without community-based components.
• The potentially explosive nature of CHIK outbreaks and the long-term sequelae of CHIKV infection make it a large public health and economic burden, especially if people cannot work due to long-lasting arthralgia or neurologic manifestations (see above).
  o Acute CHIKV infections can often affect large percentages of the population, as mentioned previously. This greatly increases the burden on hospital resources, workplaces, and the general economy of the area.
  o People with severe arthralgia cannot as easily move around or accomplish daily tasks in comparison to unaffected people.
    ▪ There was an average annual loss of over 106,000 DALYs (disability-adjusted life-years) between 2010 and 2019. Most of these DALYs are due to long-term rheumatic sequelae [6].
    ▪ Immobility also leads to higher risks in other aspects of health, and they are also less able to seek medical advice.
  o Children, especially neonates, are at risk for neurologic conditions due to CHIKV infection (see Table 1).
    ▪ Severely disabling after vertical transmission at birth and can last their whole life.

**Table 1:** Summary of epidemiology and potential indirect public health impact.

**Feature**	**Summary and evidence**
Reservoir	• CHIKV exists in both sylvatic (jungle) and urban cycles [12]. • In sylvatic cycles, non-human primate (NHP) species act as amplifier hosts and CHIKV reservoirs. Mosquito vectors include *Ae. taylori*, *Ae. luteocephalus*, *Ae. furcifer*, *Ae. neoafricanus*, and *Ae. africanus* [13], [14], [15]. • Humans can act as amplifying hosts in the urban cycle. Mosquito vectors in the urban cycle include *Ae. aegypti* and *Ae. albopictus* [12], [13]. • Other possible natural CHIKV reservoirs include: birds, rodents and other small animals [14]. • *Ae. aegypti* are moderately susceptible to CHIKV whereas the susceptibility of *Ae. albopictus* to CHIKV can vary between moderate to high [13]. • Traditionally, *Ae. aegypti* can be found in tropical and sub-tropical regions whereas *Ae. albopictus* are common in temperate regions, including several European countries and North America. However, both vector ranges are increasing geographically [13], [16], [17].

At-risk populations	• During epidemics the entire immune naïve population in the area is at risk of infection. • Populations at increased risk for more severe disease outcomes: • Individuals > 60 years (this population worldwide is expected to almost double from 12% to 22% by 2050) [9], [10], [11] • Individuals who are overweight and obese (BMI > 25; >30 kg/m^2^) [9] • Individuals with chronic disease conditions [9] • Newborn (neonatal) infants and young children [10], [18] • Pregnant women during perinatal period (49% risk of intrapartum transmission) or first trimester (rare cases of spontaneous abortion) [10] • Populations at risk for increased long-term sequelae and persistent arthralgia: females, individuals aged over 60 years, patients who had severe acute disease, and patients with other co-morbidities such as diabetes or arthritis [7].

Mortality	• Death is rare and is usually associated with an underlying condition [14]. • Estimated mortality rates in epidemics [19]: • 47.9/100,000 population in Pernambuco, 2016 • 30.1/100,000 population in Puerto Rico, 2014 • 33.8/100,000 population in Reunion Island, 2006; though some studies have shown a case fatality rate (CFR) as high as 0.1%, with this figure increasing with age and co-morbidities • CFR for at-risk subpopulations: 2.8% among neonatal infections, 0.6% among maternal-neonatal infections, 1.5% in elderly people (usually with pre-existing conditions such as diabetes and/or hypertension) [20].

Morbidity	Acute morbidity • Reported symptomatic rates vary considerably between outbreaks from as low as 15% to as high as 97%. This may, or may not, involve the contribution of viral genetic lineage. Acute disease: • High fever (above 39 °C), severe arthralgia, arthritis, headache, rash, myalgia, insomnia and exhaustion post-viremia [3], [4]. Likely to cause severe enough fatigue and pain to limit normal activities [5]. • Two phases of disease: 1. Viral phase with viremia for 5–7 days. Viremia usually resolves within 3 weeks. 2. Convalescent, non-viremic stage where symptoms typically improve in the next 10 days [3], [5], [21]. • 87–98% of cases include joint pain, which typically affects peripheral joints symmetrically [5]. • Cardiovascular events are the most common atypical clinical manifestations (approximately 40–59%) [22]. Chronic morbidity/Sequelae • On average, around 40% of people infected with CHIKV will develop chronic post-chikungunya arthritis, which may last for months or years [3], [6], [7]. • Risk of developing long-term arthralgia increases with age. More than half of the CHIKV-infected subjects from the La Reunion outbreak in 2006 over age 45 reported long-lasting muscle and joint pain [23]. • Vertical transmission is known to occur with the highest incidence associated with maternal viremia close to the time of birth. Perinatal infection can result in severe neurologic disease [3], [24]. • Average annual loss of over 106,000 disability-adjusted life years (DALYs) between 2010 and 2019. Most of these DALYs are caused by long-term rheumatic sequelae. These DALYs are drawn from the following WHO regions: African, Americas, Eastern Mediterranean, European, Southeast Asian, and Western Pacific [6]. • One of the most important public health impacts of vaccination would be to prevent or reduce CHIK-associated chronic morbidity.

Geographical and seasonal distribution	• Poor diagnostics and the similarity of early clinical disease to other co-circulating arboviral infections (e.g., dengue) limit data on CHIKV incidence [14], [25], [26], [27]. • CHIKV infections have been detected in multiple countries on all continents, except Antarctica [28]. CHIKV is endemic in numerous countries in Africa, Southeast Asia, the Indian subcontinent, Pacific Region and in the (sub) tropical regions of the Americas which bear the greatest burden of disease [29]. • Warm and wet conditions are favorable for proliferation of mosquitoes and increase transmission of CHIKV. But the virus can continue transmission throughout the year at lower rates [30]. • Outbreaks are rapid and sporadic, and usually unpredictable [3]. • Large epidemics often affect 50–70% of the population in the afflicted area [3].

Gender distribution	• Females have an increased risk of chronic CHIKV joint pain. An odds ratio of 1.68 when compared to males [31]. • No evidence of transmission via breast-feeding reported to date [32]. • Vertical transmission from viremic mother to neonate at birth carries a high risk of neonatal infection and subsequent neurological or other sequelae [3], [32].

Socio-economic status vulnerability(ies) (equity/wealth quintile)	• Socio-economically deprived areas and LMICs were more likely to have more infections and more severe outcomes [9]. • The following factors contribute to a higher burden of disease: Temperature in the area in the previous month preceding the infection, areas of low altitude for housing, rainfall in the month preceding the infection, obesity, and diabetes [9]. • Low socioeconomic status is related to decreased awareness of outbreaks; therefore, decreasing preventive care utilization and decreasing access to care in case of infection [33]. • CHIKV is present in both rural and urban areas. However, there are major health disparities in low-income neighborhoods due to decreased surveillance and low sanitation services, which lead to an increase breeding sites for mosquitoes [33]. • Areas lacking vector control and having an immunologically naïve population are particularly susceptible to CHIK outbreaks.

Natural immunity	• People of any age can be infected with and make an immune response against CHIKV. One exception may be neonatal infants, but they may have passive protection from their mothers, if the mother has been previously infected. • Neutralizing antibodies are likely a correlate of protection. Passive transfer of antibodies in mice protects against infection, mortality, and chronic infection [3], [34], [35], [36]. • Initial humoral immune response occurs at the early convalescent stage (10 days post-illness onset) [37]. • Long-lasting immunity (possibly lifelong) including neutralizing antibodies develops following CHIKV infection (similar pattern to other arboviruses) [3], [38]. • Neutralizing antibodies primarily target E2 protein [3]. • Cytotoxic CD8 T cells are elevated during mid- to late stages of acute infection and can be seen throughout chronic CHIK infection [37]. • Human T cells recognize several CHIKV encoded proteins including capsid, E2, and nsP1 [3].

Pathogenic types, strains, and serotypes	• CHIKV is a member of the family *Togaviridae,* genus *Alphavirus,* Semliki Forest virus complex. • Positive-sense, single-stranded RNA genome of 11.6 kb. • 3 genotypes (also sometimes known as “lineages”): West African (WA), East/Central/South African (ECSA), and Asian • All known strains are in a single serotype [3]. • Some strains/genotypes differ slightly in antigenic properties and neutralization but vaccines are still expected to be cross-protective [3]. • WA genotype is largely responsible for enzootic transmissions and is associated with small outbreaks in West African countries [3]. • ECSA genotype can be significantly attributed to the largest CHIKV epidemics on record. This genotype has spread to new regions leading to urban epidemics [3], [39]. • Asian genotype is responsible for the most outbreaks in Americas [40]. • However, ESCA has also been shown to be present in Brazil, Paraguay and Argentina [25]. • The Asian genotype is thought to cause less severe disease in humans than other genotypes and is more likely to result in asymptomatic infection [41]. • Most outbreaks in Africa, Asia and Europe over the past 20 years are associated with the ECSA genotype– the only exceptions being Asian lineage outbreaks in Indonesia, Philippines [25]. • Indian Ocean Lineage (IOL): a distinct subtype of ECSA that emerged in 2004. Mutations in this subtype facilitated spread by *Ae. albopictus.* (Responsible for recent outbreaks in the Indian Ocean basin and SE Asia) [3]. Preclinical studies in NHPs suggest that a vaccine targeting the IOL strain induces neutralizing antibodies against the Asian strain [42].

*Potential indirect impact*	

Anti-microbial resistance (AMR) threat	• Prescribing antibiotics as a treatment for acute fever is unnecessary for CHIKV infection as the disease is caused by a virus. It is important to increase awareness on CHIKV transmission and outbreaks to reduce the AMR threat [43], [44].

Epidemic and outbreak potential	• The emergence of CHIKV in the Americas in 2013–2014 indicates that there is high potential for other outbreaks or public health emergencies in immune naïve populations with the presence of the mosquito vectors [3]. • Outbreaks and epidemics continue to occur in various regions sporadically. For example, in February 2019, an outbreak of CHIKV disease was officially declared by the Government of Congo. Over 6,000 cases occurred between January and April [45]. • Lack of simple diagnostics for CHIKV means that outbreaks are difficult to detect until they become serious. • Outbreaks 2004–2009 also show the epidemic potential of CHIKV. In this time period, there were over 2 million cases in the Union of Comoros, La Reunion Island, and in southern India [3]. • Travel-related spread is also a concern. Typically, these are only imported cases or with limited local spread. However, the first autochthonous spread of CHIKV in Europe occurred in 2007, with ∼ 200 cases in the Region of Emilia-Romagna in the northeast of Italy. Subsequently, outbreaks have been seen in southern France, and Italy in 2017 [3], [46]. • CHIKV is identified by both government and non-government agencies as a priority pathogen. • CHIKV is part of the CEPI’s priority pathogen list [47]. • Currently, CHIKV is not on the WHO annual review of diseases prioritized under the Research and Development Blueprint.

Transmission route/potential	• Bites by CHIKV-infected *Ae. aegypti* and *Ae. albopictus* [3]. • Vertical transmission to neonates at birth when mother is viremic [3], [48]. • Reports of transmission of CHIKV via sexual intercourse. CHIKV RNA detected in semen even after 30 days post symptom onset [49].

Acquired/herd immunity	• Because large percentages of populations become infected very quickly in outbreaks/epidemics, herd immunity also forms quickly in these areas with a resultant rapid drop in incidence [3]. • Neutralizing antibodies are likely sufficient for protection (correlate of protection) [3]. • CHIK herd immunity is seen at the ∼ 50% population percentage (in Americas), leading to a decrease in new outbreaks for the current cohort [50].

Co-associated morbidity/mortality	• Comorbidities among 2,773 CHIKV patients with severe outcomes [51], • Hypertension (31.3%; 95%CI) • Diabetes (20.5%; 95%CI), • Cardiac diseases (14.8%; 95%CI) • Asthma (7.9%; 95%CI) • Compared to their younger counterparts, in the CHIKV patients over 50 years of age who had severe outcomes, there was a 4- to 5-fold significant increase in the prevalence in those individuals who had diabetes, hypertension and/or cardiac diseases [51]. • During the Brazilian outbreak in 2016, neonatal encephalitis was observed as a consequence of vertical transmission [48].

*Economic burden*	

Health facility costs/out of pocket costs/productivity costs	• CHIKV can cause more burden than other arboviruses (such as dengue), particularly in the Americas. This is partially due to the long-term sequelae of CHIKV [6]. • In the WHO’s Region of the Americas (AMRO) alone, CHIKV resulted in a disease burden amounting to over 158,000 DALYs annually [6]. • In the 2006 outbreaks in India, the estimated economic cost was US$ 8.6-US$ 17.3 million [52]. • Arthralgia secondary to the CHIKV outbreak in La Réunion in 2005–06 (750 000 inhabitants at that time) has resulted so far in an estimated total cost of up to 34 million Euros per year [53].
• There is very limited published data on the burden of disease and quality of life as affected by CHIKV outbreaks.
  o These measurements are easier to collect in higher income areas, which are typically not as negatively impacted by CHIKV outbreaks as are lower income areas.
  o This makes it difficult to construct health economic models in these areas and to establish the actual impact of CHIKV infections.
• CHIKV has a high epidemic potential.
  o Outbreaks in 2004–2009 and 2014–2015 demonstrated the epidemic potential of CHIKV. For example, there were over 2 million cases in the Union of Comoros, La Reunion Island, and in southern India during 2004–2009.
• Other types of transmission that need to be further studied:
  o There have been reports of transmission of CHIKV via sexual intercourse. CHIKV RNA can sometimes be detected in semen, even after 30 days post symptom onset. Whether or not this represents infectious virus is not known [49].
  o There is potential for vertical transmission during pregnancy and at birth, but it is unclear how high the risk is.
    ▪ Most CHIKV infections that occur during pregnancy will not result in the virus being transmitted to the fetus. There have been rare reports of spontaneous abortions following CHIKV infection in the mother, however. The highest transmission risk appears to be when women are infected during the intrapartum period. The vertical transmission rate is as high as 49% during this period. Infants are typically asymptomatic at birth and then develop fever, pain, rash, and peripheral edema. Those infected during the intrapartum period may also develop neurologic disease (e.g., meningoencephalitis, white matter lesions, brain swelling, and intracranial hemorrhage), hemorrhagic symptoms, and myocardial disease. Laboratory abnormalities included raised liver function tests, reduced platelet and lymphocyte counts, and decreased prothrombin levels. Neonates who suffer from neurologic disease often develop long-term disabilities. There is no evidence that the virus is transmitted through breast milk.
    ▪ Vertical transmission as a result of maternal-fetal blood exchange at the time of birth when the mother is viremic can result in severe outcomes for the neonates, such as lethality or severe disability.
    ▪ CHIKV genomes have been detected in human breast milk, but it is unclear whether this represents infectious virus [56].
    ▪ Antepartum vertical transmission is theoretically possible but there is little evidence that it occurs [57].
• More epidemiological surveillance, modeling, and economic burden data are needed to better estimate the true economic impact of CHIKV infections.

## 2 Potential target populations and delivery strategies

There are multiple potential target populations for CHIK vaccines, and each will require different delivery strategies. Table 2 presents these considerations. The development of a Target Product Profile (TPP) for Chikungunya vaccines may provide additional detail on priority target populations and delivery strategies.

**Table 2:** Overview of potential target and key population(s) and associated delivery strategy(ies).

**Target and key population(s)**	**Delivery strategy(ies)**
General adult population in LMIC endemic areas	• Burden of CHIK in the general population in tropical and sub-tropical areas where the mosquito vectors are prevalent is due to loss of DALYs (106,000 average annual loss between 2010 and 2019), compared to CFR (0.1% for the general population) [6], [14]. • Vaccination campaigns should be considered as a potential strategy in areas where the mosquito vectors are found abundantly, starting in areas that have had a history of CHIKV or are close to areas with a history of CHIK outbreaks. • Optimal timing for vaccination campaigns may occur before or after seasons associated with known higher transmission intensity. Higher transmission months may vary based on context/ country. • As with most adult vaccines and campaign vaccines, single-dose vaccine would be a preferential choice to increase accessibility and uptake of the vaccine. To date, single-dose vaccine candidates are either live or recombinant-based vaccines [58], [59]. • Inactivated, VLP, and recombinant vaccines often have higher thermostability than live vaccines and could be preferred for campaigns in rural or low-income areas but require multiple doses to induce protective immunity. • Outbreak response: similar to outbreak response with Ebola vaccine candidates, vaccines can be quickly delivered to areas with outbreaks by utilizing existing resources within WHO, UNICEF, IFRC, MSF, etc. [60]. • Immunization schedule: delivery to adults should occur when outbreaks are detected and throughout the year in endemic areas. If vaccines are available for the pediatric age indication, delivery to children could be incorporated within the childhood vaccination schedule if it is safe to administer with other vaccines.

General population (working age) in MIC/HIC endemic areas	• Vaccine delivery should be available to all eligible members of the population, with a focus on high-risk groups (older, females, travelers, etc.). • Most accessible site for vaccine delivery would be primary care visits, which allows for identifying high-risk individuals. Other methods, including family planning clinics, immunization campaigns, and school programs for adolescents (in available countries) would increase vaccination uptake. • Preferred candidates for MICs/HICs are single-dose vaccines. These would be especially helpful during sporadic outbreaks, as they would provide fastest immunization across all candidates. • Countries beyond the tropics and sub-tropics that have been affected by outbreaks in the past in certain areas, or have the mosquito vectors, may consider vaccination.

Older individuals with pre-existing conditions (primarily cardiac, liver, or kidney) in endemic areas	• CFR is estimated to be 1.5%, with increased risk for those with pre-existing medical conditions [60]. o 40 – 70 years, increased risk for severe disease and mortality. o > 65 years, increased risk for central nervous system disease. • Immunogenicity and effectiveness may be lower in older individuals. Vaccination may nevertheless be indicated as older individuals are at higher risk of severe disease and sequelae, provided that such vaccines are safe in that age group. • Immunization schedule: Adults can receive the vaccine when it is appropriate or during an outbreak.

Travelers to endemic areas	• All travelers, and especially those who are at risk of more severe disease outcome, should consider vaccination prior to travel to endemic areas (Africa, Southeast Asia, Pacific Region, and subtropical areas of the Americas). Vaccination should be recommended during (or prior to) CHIKV season [61]. • The risk of exposure to CHIKV depends on the itinerary, duration of travel, season, and activities undertaken during travel and/or within endemic areas [62]. • Delivery could be dependent on identification of individuals at-risk. Further, those planning travel to endemic areas should consider vaccination 2 months prior to departure (or shorter, depending on vaccine efficacy and immunogenicity trials).

Women of child-bearing age and newborns in endemic areas	• Animal studies should be recommended to look for evidence of teratogenicity and fertility. • A package of such safety data should be routine for this population. • Female sex is a risk factor for chronic disease, especially for chronic arthritis and arthralgia [31], [63]. • CFR is estimated to be 0.6% for maternal infection and 2.8% for infant infection [64]. o < 3 years infant: increased risk for central nervous system disease [20]. o Cohort studies show 15.3% chance for vertical transmission [20]. o To date, there are no clinical trials in infants. • To achieve rapid immunization prior to late gestation and/or birth, there should be a preference for non-replicating candidates **and** single-dose options for pregnant women. • Immunization schedule: ideally administered prior to pregnancy. If not, trials in pregnant women and infants will need to be carried out and should focus on low-risk vaccines such as those mentioned previously.

Secondary MICs/HICs Targets: General population outside of endemic areas	• Vaccine delivery should be available to all eligible members of the population, with a focus on high-risk groups (elderly, females, travelers, etc.). • Most accessible site for vaccine delivery should be primary care visits – also allows for identifying high-risk individuals. As CHIKV continues to spread its transmission towards areas outside of tropical weather, and moves to temperate and sub-tropical conditions, it would be important to re-evaluate endemic areas and access to vaccine outside of CHIKV season.

## 3 Chikungunya and its consideration as a public health priority by global, regional or country stakeholders

Stakeholders engaged in CHIK vaccine research, development, and advocacy are primarily concentrated in the regions in which CHIKV is endemic, as well as within global/ multi-lateral organizations with mandates that include infectious disease vaccine development. Table 3 presents information on key stakeholders for CHIK vaccines.

**Table 3:** Overview of non-commercial stakeholders engaged, their interest and potential demand. N/A: Not available.

**Stakeholders engaged**	**Summary of position/interest**	**Potential activities**
WHO regions	PAHO has published a preparedness and response plan for CHIK outbreaks, including the surveillance of CHIK in the Americas, training of health workers to identify and manage cases of CHIK, and the implementation of vector control strategies. Vaccination against CHIKV would free up resources for PAHO to focus on other diseases such as Zika and other health issues [65]. WHO Global Arbovirus Initiative [66]: integrated approach that aims to collate the crucial components of the detection, prevention, and control of arboviruses including dengue, Zika chikungunya and yellow fever [67].	N/A

Gavi	Gavi helps by improving the access to new vaccines in the lower income countries. CHIK was previously reviewed as part of the 2018 Vaccine Investment Strategy (VIS). CHIK vaccine candidates were then included in the category of vaccines under consideration for epidemic preparedness and response for which close monitoring would be done through “living investment” analysis until an investment case could be put together for approval by the Gavi board [68], [69].	• New Vaccine Investment Strategy (VIS) cycle to begin in 2024, in which CHIK vaccine has been recommended for inclusion. • A majority of the early adopter countries and countries generally affected by CHIK (especially in South America) are currently not eligible for Gavi support and will require additional discussions with Gavi to discuss funding possibilities. Gavi’s ongoing 5.0 strategy had flagged appetite for providing support to middle-income countries for which CHIK could be an adequate entry point.

Coalition for Epidemic Preparedness Innovations (CEPI)	CEPI is currently funding advanced development and clinical testing of two CHIK vaccine candidates – Valneva VLA1553, and Bharat Biotech and International Vaccine Institute inactivated vaccine. CEPI is also expected to support the licensure of VLA1553 in Brazil plus the development, manufacture, and distribution of the Bharat/IVI inactivated CHIK vaccine in low- and middle-income countries [70], [71], [72].	N/A

Government agencies (CDC, ECDC, NCVBDC, Africa CDC)	Regional government agencies are involved in the surveillance, preparedness, and communication of the CHIK in the given region.	No published positions papers from any government yet.

Research experts	Several academic, government and commercial research laboratories are involved in the development of CHIK vaccines using various platform technologies [73], [74].	N/A

## 4 Existing guidance on preferences/preferred product attributes for vaccines against Chikungunya

At present, there are no publicly available Preferred Product Characteristics documents (PPCs) or TPPs, other than those developed by vaccine developers. These are typically developed in order to determine the characteristics identified in Table 4. However, as vaccine development has advanced, these may no longer be necessary for CHIKV.

**Table 4:** Summary of existing guidance on preferences for product attributes of vaccines intended for use in LMICs.

**Product attribute**	**Minimal characteristic, if described**	**Preferential characteristic**	**Publishing entity**
Indication	N/A	N/A	N/A

Target population(s)	N/A	N/A	N/A

Outcome measure(s) and target efficacy	N/A	N/A	N/A
Safety profile	N/A	N/A	N/A

Number of doses and schedule	N/A	N/A	N/A

Route of administration	N/A	N/A	N/A

Duration of protection	N/A	N/A	N/A

Co-administration with other vaccine	N/A	N/A	N/A

Product stability and storage	N/A	N/A	N/A

Vaccine presentation	N/A	N/A	N/A

## 5 Vaccine development

### 5.1 Probability of technical and regulatory success (PTRS)

There are currently no licensed vaccines for CHIKV. However, diverse target populations exist (LMIC and HIC, younger and older populations, etc.) and could be utilized to study vaccine efficacy.

Differences in vector control and other confounding factors in LMICs may affect how efficacy is measured for vaccine clinical trials.

Since neutralizing antibodies are likely to be used to calculate a correlate of protection, both active and passive vaccination trials should be undertaken. Given that there are multiple genotypes of CHIKV, there is also the potential to evaluate how well different vaccines can protect against all genotypes. This may also give insight on how to vaccinate other diseases with similar genetic variability, including other alphaviruses. Table 5 presents details on parameters that may inform vaccine development from the perspective of CHIKV.

**Table 5:** Overview of parameters that inform scientific feasibility of developing an effective vaccine for LMIC public market use.

**Parameter**	**Issues and evidence**
Diagnosis/case ascertainment	• CHIKV infection is considered when patients present with severe polyarthralgia and fever after traveling to or living in areas where CHIKV is present. Laboratory diagnostic techniques include detection of the virus, viral RNA, and/or IgM against the virus in the patient serum or plasma [14]. • Early symptoms of CHIK overlap those of multiple other acute febrile diseases, including dengue and malaria that frequently co-circulate in the same geographic areas. This poses challenges for the rapid diagnosis and treatment of CHIK [75]. • PAHO guidelines indicate mucosa bleeding and signs of arthralgia are the best indicators for possible CHIKV infection (but there is the potential to be confused with dengue) [75]. • CHIKV isolation by cell culture is the “gold standard” method to confirm infection. However, it is time-consuming and requires specialized facilities. Also, culture is only sensitive during the early stage (viremic phase) of the disease, after which the sensitivity drops [76]. As such, currently, RT-PCR is the definitive method for virus identification by detecting viral RNA. • CHIKV IgM antibody capture ELISA is a widely used diagnostic tool. IgM antibodies persist after the viremic phase of the disease and typically reach their highest levels 3–5 weeks after the onset of illness and can last up to two months post-infection [76]. • IgM/IgG ELISA with purified viral antigen is recommended due to its high sensitivity to CHIKV and low cross-reactivity with other alphaviruses. Though IgG may also indicate a past CHIKV infection [77]. • An RT-PCR or RT loop-mediated isothermal amplification (LAMP) test for the detection of viral RNA could be developed, though it involves the use of expensive reagents and equipment (though LAMP would not require a thermocycler) and only works during the viremic phase. There is no solid evidence that the specificity and sensitivity of these methods of viral genome detection will be limited by the existence of multiple CHIKV genotypes.

Biomarkers/ Correlates of risk and/or protection	• Studies in animal models, and limited clinical studies, show CHIKV-specific neutralizing antibodies can reduce viral load and protect against clinical symptoms, including the development of severe disease [3], [34], [35], [36]. • Passive transfer studies with neutralizing antibodies have shown protection in animal models, but information is very limited on challenge with CHIKV strains representing different genetic lineages. • Absence of a definitive clinical or laboratory biomarker(s) to differentiate between CHIKV and other acute febrile illnesses complicates diagnosis. • Presence of CHIKV-specific IgM and IgG antibodies after the acute phase of infection can serve as a biomarker for potential of recurrent infection. Neutralizing antibodies against CHIKV primarily target the E2 protein [3]. • Neutralizing antibodies are considered a correlate of protection, but an adequate threshold of neutralizing antibody titers has not been established [3]. • Cytotoxic CD8 T cells are elevated during mid- to late stages of acute infection and can be seen throughout chronic CHIK infection but would be a difficult biomarker to standardize [37]. • Increased levels of IL-6 and IL-1β and decreased RANTES levels are associated with severe CHIKV disease [78]. • High levels of IL-6 and GM-CSF also coincided with persistent arthralgia [79].

Sero-epidemiological data	• Pre-existing alphavirus immunity may interfere with neutralizing antibody responses to certain vaccines, especially those that require vaccine replication (i.e., LAVs). This requires further studies. • Themis Biosciences/Merck have shown that pre-existing immunity to the measles virus vaccine vector did not affect the immunogenicity of their vaccine candidate, MV-CHIK [80]. Further research on vaccine vector pre-existing immunity would be required for other vaccine candidates that utilized a viral vector.

Clinical endpoints	• Another endpoint could be protection from any febrile illness (RT-PCR confirmed) due to CHIKV infection, which can be monitored during a clinical trial. • Primary clinical endpoints include protection from severe CHIK (neonatal chikungunya, neurological manifestation, multiorgan failure which lead to deaths specially in people with comorbidities) and from severe sequelae, including persistent arthralgia and neurological effects. • Secondary endpoints: Reduced viremia. During a clinical trial, viremia in a CHIKV-infected patient could be measured by quantifying the amount of virus (viral titration, if specialized facilities are available) or amount of viral genomic RNA in the serum or blood. A reduction in viremia would reduce subsequent transmission of CHIKV to mosquitoes and would theoretically help control outbreaks.

Controlled Human Infection Model (CHIM)	There is no current Controlled Human Infection Model (CHIM) for CHIK. If one is developed, the limitations listed below must be considered. • While CHIKV infection rarely causes fatal disease, the high rate of recurrent disease/sequelae, including severe arthralgia, makes planning of CHIM studies difficult and ethically challenging. • There are no specific licensed “rescue” treatments for CHIK disease, further increasing the potential risk of CHIMs. • If human challenge studies are to be accomplished, live-attenuated vaccine CHIKV may be used instead of more virulent wild-type viruses. However, CHIMs utilizing LAVs for human challenge would primarily study protection from viremia and possibly fever, not protection from severe disease and arthralgia. • Due to the above considerations, CHIMs are clinically possible but realistically unlikely to occur at the present time.

Opportunity for innovative clinical trial designs	• Multiple diverse target populations exist (LMIC versus HIC, younger versus older populations, etc.) and could be utilized to study vaccine efficacy. • Differences in vector control and other confounding factors in LMICs may affect how efficacy is measured for vaccine clinical trials. • Since neutralizing antibodies are likely to be a correlate of protection, both active and passive vaccination trials could be undertaken. • Given that there are multiple genotypes for CHIKV, there is a great opportunity to study how well different vaccines can protect against all genotypes. This may also give insight on how to vaccinate for other diseases with similar genetic variability, including other alphaviruses. • Due to the sporadic nature of CHIK outbreaks, ring vaccination, and contact tracing can be utilized as innovative clinical trial designs. • Seamless phase II/III clinical trials can be designed to expedite vaccine licensure and to target populations during outbreaks [81]. • Sample size re-estimation, adaptive design, and Bayesian approaches may be utilized before and after interim clinical trial analyses as the sporadic nature of outbreaks and endemic infections may make it difficult to predict incidence rates during the trials [81].

Regulatory approach(es)	• Traditional approval pathway based on predicted protection against CHIKV infection is dependent on clinical disease end-point. However, successful performance of randomized controlled clinical endpoint efficacy trials is currently considered unlikely due to the irregular and unpredictable nature of CHIK outbreaks [82]. • Other approval pathways include accelerated approval (US FDA) or conditional approval (EMA) that is based on predicted clinical benefit and animal approval which is based on disease end-points in animal models of infection [82]. • At present, the most likely pathway to regulatory approval is a non-traditional approach utilizing a combination of data from sero-epidemiological studies and from animal challenge studies (using passively transferred sera from human vaccinees), which would facilitate identification of an immune marker reasonably likely to predict vaccine effectiveness [82]. • There is a potential for WHO Emergency Use listing if there was a Public Health Emergency of International Concern or similar temporary authorization by national regulatory authorities, e.g., US FDA Emergency Use Authorization.

Potential for combination with other vaccines	• There is little information on interactions between CHIK vaccine candidates and other vaccines and/or induction of immune responses. • Different vaccine platforms (e.g., live attenuated, inactivated whole virus, VLP) have been utilized for candidate CHIK vaccines and so combination studies with licensed vaccines would need to be evaluated on a case-by-case situation. • Live attenuated vaccines are rarely licensed to be combined with other live attenuated vaccines without extensive testing.

Feasibility of meeting presentation and stability requirements	• The vaccine candidates for CHIK vary in their stability requirements from −80 to −10 °C, as of current trials. They also vary in expiration times with and without reconstitution protocols. • Stability and storage requirements are assessed for each vaccine in pre-clinical trials. • WHO preferred product characteristics for a CHIK vaccine have not been described.

Vaccine platform	Vaccine candidates by platform, further detail in Table 6. • Live attenuated; e.g. VLA1553 • Live virus vector; e.g. MV CHIK 202 (recently discontinued [83]) • Defective viral vector; e.g. ChAdOx1 • Inactivated; e.g. BBV87, HBI/CG/I/2020/001.02.00 • Virus like particle; e.g. PXVX0317 • mRNA; e.g. VAL-181388

Large scale Manufacturer capacity / interest	• Vaccine manufacturers have varying manufacturing capabilities. Manufacturers are listed in Table 6 with vaccine candidates. • Vaccine platforms should be suitable for large-scale production: mRNA large-scale production can be increased as shown by the COVID-19 pandemic. Live attenuated vaccines and inactivated vaccines will require cell culture to grow the virus. Subunit vaccine production can typically be scaled up adequately. • Information on ease and scale of manufacturing are not available in the public domain.

### 5.2 Overview of candidates in the clinical pipeline

**Fig. 1:**
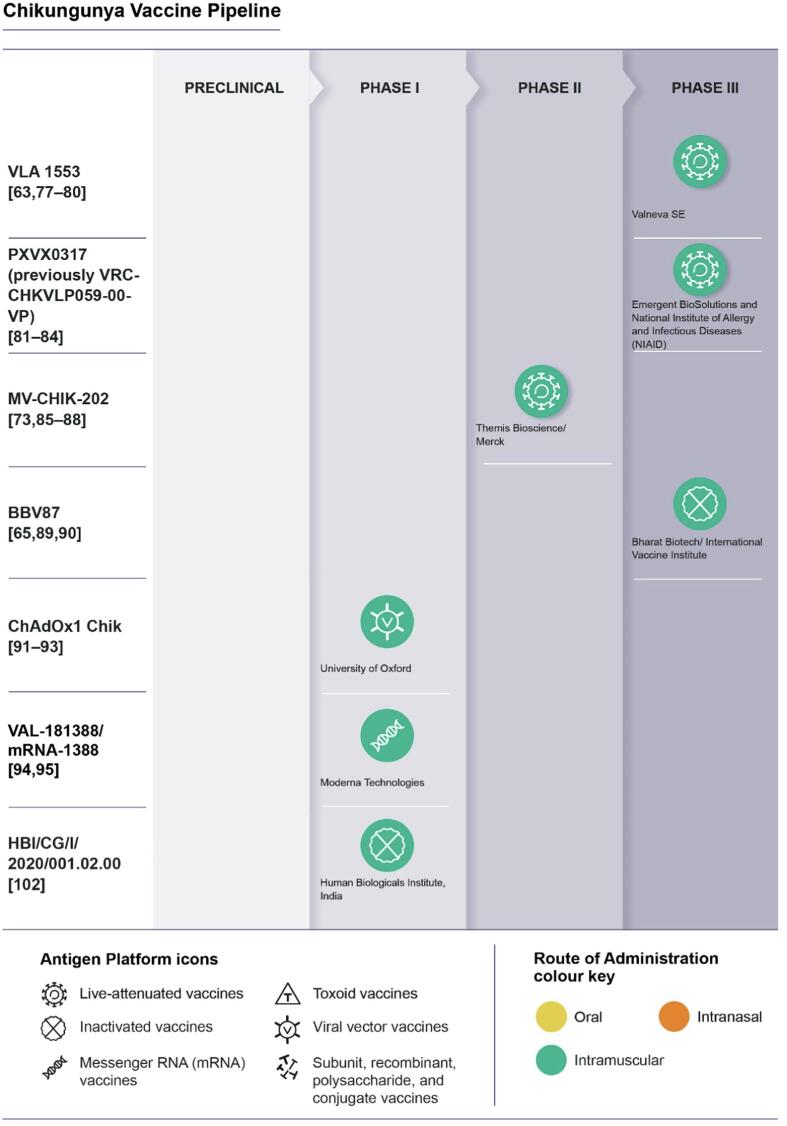
Overview of vaccine candidate in clinical trials.

As of July 1, 2023, there are no licensed vaccines against CHIKV. However, there are several vaccine candidates in clinical evaluation, including Valneva’s VLA1553 and Bavarian Nordic (acquired from Emergent BioSolutions [84]) PXVX0317 in phase III, Bharat Biotech’s BBV87 in phase II/III, Themis/Merck’s MV-CHIK-202 in phase II (recently discontinued [83]), and Oxford University’s ChAdOx1 Chik and Human Biologicals Institute’s HBI/CG/I/2020/001.02.00 and Moderna Technologies VAL-181388 in phase I (see Fig. 1).

These most advanced CHIKV vaccine candidates are based on multiple strategies/ platforms (references in Table 6):

• VLA1553 is a live-attenuated vaccine (LAV) that contains the attenuated Δ5nsP3 virus strain with deletion in the replicase region.
• PXVX0317 is a virus-like particle (VLP) based vaccine composed of E1, E2 and capsid proteins from the 37,997 strain of CHIKV.
• MV-CHIK-202 is a recombinant, live-attenuated vaccine containing the Schwarz vaccine strain of measles virus (MV) engineered to express the CHIKV structural proteins, E1 and E2.
• BBV87 is an inactivated viral vaccine containing the CHIKV strain derived from ECSA genotype.
• ChAdOx1 Chik is a recombinant virus-vectored vaccine that uses a chimpanzee adenovirus expressing CHIKV structural proteins (C-E3-E2-6 k-E1).
• VAL-181388 is a mRNA-based vaccine that contains a single mRNA encoding the CHIKV structural polyprotein (C-E3-E2-6 k-E1).

**Table 6:** Overview of vaccine candidates in clinical trials.

**Candidate**	**Antigen Platform**	**Developer/ manufacturer**	**Phase of development, population and location**	**Route of administration, no. of doses, schedule**	**Adjuvant**
VLA 1553 [70], [85], [86], [87], [88]	Live-attenuated CHIKV with deletion in nsP3 replicase region.	Valneva SE	Phase III (VLA1553-301) Age 18–45 years; United States. Completed. Phase III (VLA1553-302) Age 18–45 years; United States; Lot to lot consistency study. In Progress. Phase III (VLA1553-321) Age 12–17 years; Brazil In Progress. Recruiting.	Single intramuscular immunization, Dose 1 x10E4 TCID50. Placebo: phosphate buffered saline	None

PXVX0317 (previously VRC-CHKVLP059-00-VP) [89], [90], [91], [92]	Virus-like particle (VLP) Containing E1, E2 and capsid proteins from CHIKV strain 37997.	Bavarian Nordic (acquired from Emergent BioSolutions [84] and National Institute of Allergy and Infectious Diseases [NIAID])	Phase III Ages 12––64 years; United States. In Progress, Recruiting.	Single intramuscular immunization Dose (40 µg) Placebo: Vaccine diluent.	Alhydrogel® (2%)

MV-CHIK-202 [80], [93], [94], [95], [96] (Recently discontinued [83])	Live-attenuated measles-virus vector containing CHIKV, E1 and E2.	Themis Bioscience/ Merck	Phase II Adults 18–55 years; Austria and Germany. Completed. Results published. Phase II Ages 18–55 years United Kingdom. Completed. Phase II Ages 21–50 years; Puerto Rico. Completed. Phase II Ages 21–65 years; Puerto Rico. Completed.	Single or two intramuscular immunizations 28 days apart. Doses 5xE4 ± 0.5, and 5xE5 ± 0.5 log TCID50. Placebo: saline and/or approved MMR vaccine (Priorix ®)	None

BBV87 [72], [97], [98]	Inactivated CHIKV strain derived from an East, Central, South African (ECSA) genotype.	Bharat Biotech/ International Vaccine Institute	Phase II/III Ages 12–65 years; Panama, Columbia and Thailand. In progress. Phase II/III Ages 12–65 years; India. In Progress.	Two intramuscular immunizations 28 days apart Doses 20 µg and 40 µg tested. Placebo: Sodium Chloride Injection IP/BP 0.9% (Normal Saline)	Alum (0.25 mg)

ChAdOx1 Chik [99], [100], [101]	Chimpanzee adenoviral vector expressing Capsid, E3, E2, 6 K, and E1 of CHIKV.	University of Oxford	Phase I Ages 18–50 years; United Kingdom. Complete; Results published. Phase Ib Ages 18–50 years; Mexico. In Progress.	Phase I Single intramuscular immunization Doses 5x10^9^, 2.5x10^10^, 5x10^10^ virus particles tested No placebo. Phase Ib: Single intramuscular immunization Doses 5x10^9^, 2.5x10^10^, 5x10^10^ virus particles of ChAdOx1 Chik, ChAdOx1 Zika, or a combination. Placebo: isotonic saline solution (0.9%)	None

VAL-181388/ mRNA-1388 [102], [103]	mRNA encoding the CHIKV structural proteins.	Moderna Technologies	Phase I Ages 18–49 years; United States. Study complete Results not yet posted.	Two intramuscular immunizations week 0 and week 4 Doses 25 μg, 50 μg or 100 μg tested Placebo: saline	Unknown

HBI/CG/I/2020/001.02.00 [105]	Inactivated virus	Human Biologicals Institute, India	Phase I Ages 19–49 years; India. In progress.	Two or three intramuscular immunizations (days 0–28 or 0–28-56) Dose unknown Placebo: Saline (0.9% w/v)	Unknown

## 6 Health impact of a vaccine on burden of disease and transmission

There is currently no dedicated antiviral drug against Chikungunya virus. Recommendations for protection thus focus on infection control through preventing mosquito proliferation. Therefore, an effective vaccine against Chikungunya virus would likely carry a significant impact on disease burden and prevention control (see Table 7, Table 8, Table 9, Table 10).

**Table 7:** Overview of studies to measure disease burden and transmission.

**Policy question**	**Assessment method/measure**	**Outcomes/interpretation**
To characterize the global epidemiology of CHIK and inform vaccine development [25]	Systematic literature review and assessed CHIK epidemiological trends from 1999 to 2020.	• Identify substantial gaps in epidemiological knowledge, especially granular data on disease incidence and age-specific infection rates. • Diversity of methodologies and study designs, and reflects a lack of standardized procedures to characterize this disease • Challenges to conduct vaccine efficacy trials due to limitations in available epidemiological data and disease unpredictability. • Better understanding of CHIK dynamics with appropriate granularity and better insights into the duration of long-term population immunity are critical to assist in the planning and success of vaccine development efforts pre- and post-licensure.

To estimate the global burden of CHIK between 2010 and 2019 [6]	Systematic review of published literature and surveillance records to estimate global burden of CHIK between 2010 and 2019, including annualized DALY estimates.	• CHIK caused an annual disease burden of 106,000 DALYS between 2010 and 2019. • Substantially higher burden in the Americas in comparison to other WHO regions. • Long-term rheumatic sequelae provided the largest DALY component for CHIK burden. • CHIK causes significant morbidity and transmission-blocking strategies, including vector control and vaccine development remain crucial priorities in reducing global disease burden through prevention of potentially devastating Chikungunya outbreaks.

**Table 8:** Overview of modelling studies that measure anticipated socio-economic impact of the vaccine.

**Policy question**	**Assessment method/measure**	**Additional information specific to models**	**Assumptions**	**Outcomes/ interpretation**
Quantifying the likely impact of a CHIK vaccine across Gavi-eligible countries within different epidemic scenarios (e.g., sporadic localised epidemics, widespread but infrequent epidemic, endemic transmission). NB. Scenario model selected due to uncertainty in current levels of infection across countries Internal Gavi model (personal communication from Gavi)	Simulation framework per epidemic scenario. Transmission model included to generate potential size and timings of outbreaks per scenario	Different vaccine rollout strategies will be overlaid per epidemic scenario (e.g., vaccine stockpiling, integration of vaccine into immunisation programmes). Additional analyses based on different characteristic of vaccines and role of disease surveillance	Within the model, each Gavi country will be assigned a CHIK “epidemic state” (i.e., no disease, epidemic, endemic) based on literature review and critical assessment of existing global prediction models	Key metrics per scenario include number of cases/deaths averted, DALYs averted

**Table 9:** Overview of expectations of evidence that are likely to be required to support a global / regional / national policy recommendation, or financing.

**Parameter for policy/financing consideration**	**Assumptions**	**Guidance/reports available**
Burden of disease	• Incidence, long-term sequelae, DALY, magnitude of outbreaks, and socio-economic impacts should be quantifiable and understood.	Burden of disease is unpredictable, varies over time, and is difficult to quantify; more data are needed in this area.

Value of the vaccine in the context of other control measures	• Vector control can reduce the magnitude and frequency of outbreaks, but is often not sustainable nor scalable and just reactive	WHO guidance on vector control [109] WHO Global Arboviral Strategy [110]

Economic impact of the disease and potential cost savings from a vaccine	• Evidence from cost-effective studies should demonstrate differences in vaccine use versus vector control (and combination) • Evidence of socio-economic disruption is needed (e.g. from the Latin American outbreak as an example) • Modelling data should provide evidence of the magnitude of future CHIKV outbreaks	Research gaps; more evidence is needed

Vaccine price and affordability	• Dosage, regimen, and cost of goods should be amenable to affordable supply. • Favorable cost-effectiveness should be established, and cost should not be a barrier to access, including in LMIC. • Vaccine and programmatic logistics costs should be available and amenable to LMICs.	WHO Evidence Considerations for Policy Development [111]

Impact of the vaccine on antibiotic use and AMR	• Empiric treatment with antibiotics for community-acquired febrile illnesses is high	Empiric treatment with antibiotics for community-acquired illnesses has been described for dengue but not yet for Chikungunya [112]

**Table 10:** Research gaps pertaining to epidemiology, indirect public health impact, economic burden, and other considerations.

**Issue**	**Research Gaps**	**Data Needed to Address Gaps**
Epidemiology	• Burden of symptomatic acute disease and long-term chronic disease in the pediatric population < 18 years of age. • Burden of symptomatic acute disease and long-term chronic disease in the immunocompromised including HIV and pregnant populations. • Required TPP of a vaccine to diminish the outbreak of an epidemic (diminished or no viremia to eliminate vector spread). • Clinical study design to test vaccine efficacy, i.e. of a test negative design. • CHIK dynamics with appropriate granularity at smaller geographical scales – to inform possible location of efficacy studies (need = prospective cohort studies, enhanced surveillance) • Duration of population protection following an outbreak – to inform feasibility and possible location of efficacy studies (need = seroprevalence studies with participant follow-up for a sufficient time period) • Specific case definitions for co-circulating arboviruses, to decrease risk of misclassification in co-endemic areas where majority of cases are clinically diagnosed and not lab-confirmed. • Characterize cross-reactivity with other alphaviruses.	• Meta-analysis of available published or country-specific acquired data. • Prospective cohort studies. • Meta-analysis of available published or country-specific acquired data. • Prospective cohort studies. • Data modelling • Data modelling • Could be done as part of a comparative diagnostics study, if that is also a need.

Potential indirect public health impact	• Long-term productivity loss	• Data pertaining to time period of long-term morbidity • Data pertaining to productivity loss

Economic burden	• Analysis to derive cost-of-illness • Required TPP of a vaccine to diminish the outbreak of an epidemic (diminished or no viremia to eliminate vector spread) and potential impact on economic burden. • Economic burden of symptomatic acute disease and long-term chronic disease in the pediatric population < 18 years of age. • Economic burden of symptomatic acute disease and long-term chronic disease in the immunocompromised including HIV and pregnant populations.	• Direct medical costs (staff time, diagnostic tests, medical devices, clinic overheads, etc.), and future related health service costs • Direct non-medical costs (travel, accommodation, meals, etc.) • Indirect non-medical costs (productivity loss and informal care costs associated with other’s time) • Data modelling (all questions)

Modelling health impact on disease burden and transmission	• Model to predict health impact derived from CHIK vaccination • Impact of climate change on CHIKV transmission • Categorization of each country into different epidemic states (low/medium/high) using various data sources • Quantifying the public health impact of vaccination and different vaccination strategies, based on a given set of epidemic scenarios (all countries) • Stockpile requirements for routine and/or outbreak response • Locations of outbreaks for clinical trials and/or stockpile hub locations	• Age-specific infection rates and seroprevalence estimates • Age-specific incidence • Climate change models and estimates of likely resultant proliferation of relevant mosquito species. Population density (to represent urbanization) • Altitude • Chikungunya suitability/risk • Cases data • Vector data • CHIKV-specific transmission characteristics

Anticipated socio-economic impact of the vaccine	• Assess the need to translate a biomarker for a protective neutralizing antibody for one genotype to other circulating genotypes using NHP antibody transfer studies. • Well-characterized cynomolgus macaque models of infection (e.g. disease natural history studies done under high quality research conditions) using well characterized virus stocks made from strains from different regions. Datasets would demonstrate with confidence if certain strains result in differing levels of pathology (per comment above about circulating genotypes). • Models for acute vs chronic joint pathology. • Models in species other than NHP. • Good characterization of innate immune responses and cellular immune responses during CHKV infection in any model. • Return on investment analysis	• NHP antibody transfer studies to establish a biomarker of protection for each genotype. • NHP transfer studies to make use of an international Antibody standard reagent which would provide antibody concentration/basis for comparison across studies. • Assays for biomarkers of infection (validated RT-PCRs, immune response markers of interest). • Value of healthy life-year • Cost-of-illness • Vaccination costs

The health impact of the vaccine will include both direct and indirect effects [104]. The direct effect of the vaccine will lead to reduction in age-specific chikungunya incidence and prevalence in direct proportion to vaccine coverage and efficacy as well as based on the age of vaccination and duration of vaccine-derived immunity against chikungunya infection and disease. The indirect effect of the vaccine will lead to reduction in transmission and this herd effect will add to the direct effect in further reduction in chikungunya infection and disease.

### 6.1 Summary of knowledge and research gaps in modelling health impact on disease burden and transmission

See Section 10.

## 7 Social and/or economic impact of a vaccine

Although the viral infection rarely leads to patients’ deaths, Chikungunya arthralgia is extremely painful and debilitating with effects lasting from a few weeks to a few years. Thus, the economic consequences of morbidity due to CHIK adversely impact large populations engaged in subsistence farming and in other labor-intensive jobs in LMIC.

Based on a study of the 2013–2015 CHIK epidemic in the Americas, the health impact was estimated at over 39.9 million cases in the Americas alone after accounting for under-reporting [105], [106]. The total disease burden was estimated at 23.8 million DALYs along with an economic burden of US$ 185 billion from the societal perspective, with 95% of the costs attributed to chronic inflammatory rheumatism.

The economic burden of CHIK will be reduced at least in direct proportion to the vaccine coverage and efficacy based on the age of vaccination and duration of protection, and additional economic burden will be averted due to the indirect herd effects of the vaccine. There is potential for further spread of CHIKV to regions, countries, and territories that are not currently at risk of CHIKV, due to climate change, globalization, viral evolution and vector adaptation, that could lead to relatively higher health, economic, and social impact than the current estimates attributed to CHIK outbreaks [107]. Correspondingly, there is potential for CHIKV vaccines to have a relatively higher impact on reduction in health, economic, and social impact in the future.

### 7.1 Summary of knowledge and research gaps in modelling studies that measure anticipated socio-economic impact of the vaccine

See Section 10.

## 8 Policy considerations and financing

A full value of vaccine assessment (FVVA) should be conducted to illustrate the global public health rationale for developing vaccines against acute, subacute, and chronic diseases caused by Chikungunya infection, evidence synthesis of health and economic impact, and inform decision making across the continuum of vaccine development, introduction, and sustainable implementation for public health impact [108]. The evidence synthesis from FVVA will be a valuable input for policy-related activities that aim to create an enabling environment at the national, regional, and global levels for the introduction of CHIKV vaccines. This includes supporting the interaction with Strategic Advisory Group of Experts on Immunization (SAGE) advisory committees and generation of SAGE approval recommendations which pave the way for generation of regulatory strategies at the national level. The regulatory strategies will facilitate approval, recommendations for use, and introduction of CHIKV vaccines in affected and early adopter countries.

The global investment case developed in the FVVA process will serve as a valuable input in developing the financing and investment strategies. For the early adopter countries and future eligible countries, a sustainable financing mechanism must be developed in collaboration with Gavi, the Vaccine Alliance as part of their inclusion of CHIKV vaccine in the 2024 Vaccine Investment Strategy.

## 9 Access and implementation feasibility

At present, there are no publicly available Preferred Product Characteristics documents (PPCs) or TPPs, other than those developed by vaccine developers. These must be developed in order to determine the suitability of Chikungunya vaccines with respect to access and implementation feasibility.

## 10 Research gaps

As noted throughout this vaccine value profile, there are many existing gaps with respect to the information required for Chikungunya vaccine research and development. Table 10 summarizes those gaps.

## 11 Conclusion

While mortality due to CHIKV infection is low, the morbidity and overall burden of disease is high. After the acute disease phase, chronic sequelae, including persistent arthralgia are common with around 40% of people infected with CHIKV developing chronic post-CHIK arthritis, which may last for months or years. Therefore, one of the most important public health impacts of an effective vaccine would be to prevent or reduce CHIKV- associated chronic morbidity. This benefit would particularly be seen in LMICs and socio-economically deprived areas, as they are likely to have more infections and more severe outcomes.

There are several potential target populations for Chikungunya vaccines, and each will require some variance in proposed delivery strategies. At present, there are no publicly available PPCs or TPPs, other than those developed by vaccine developers. The development of these documents is critical in order to provide clarity on priority target populations, delivery strategies, and access and implementation feasibility. PPCs and TPPs can also inform clinical aspects of vaccine development.

As of July 1, 2023, there are no licensed vaccines against CHIKV. However, there are several vaccine candidates in clinical evaluation, including Valneva’s VLA1553 and Bavarian Nordic's PXVX0317 in phase III, Bharat Biotech’s BBV87 in phase II/III, and Oxford University’s ChAdOx1 Chik and Human Biologicals Institute’s HBI/CG/I/2020/001.02.00 and Moderna Technologies VAL-181388 in phase I.

It is important that a full value of vaccine assessment (FVVA) be conducted to illustrate the global public health rationale for developing vaccines against acute, subacute, and chronic diseases caused by CHIKV infection, evidence synthesis of health and economic impact, and inform decision making across the continuum of vaccine development, introduction, and sustainable implementation for public health impact [110]. The evidence synthesis from FVVA will be a valuable input for policy-related activities that aim to create an enabling environment at the national, regional, and global levels for the introduction of CHIKV vaccines. This includes supporting the interaction with SAGE advisory committees (PDVAC, IVIR-AC, NSB) and generation of SAGE approval recommendations which pave the way for generation of regulatory strategies at the national level. The regulatory strategies will facilitate approval, recommendations for use, and introduction of CHIKV vaccines in affected and early adopter countries.

The global investment case developed in the FVVA process will serve as a valuable input in developing the financing and investment strategies. For the early adopter countries and future eligible countries, a sustainable financing mechanism must be developed in collaboration with Gavi, the Vaccine Alliance for their review and inclusion of CHIKV vaccine in the next Vaccine investment Strategy planning cycle, which starts in early 2024.

## Author agreement

The authors declare that this is original work which has not been published before, and that all authors have agreed to the submitted paper.

## Funding

This work was supported by the World Health Organization. KA is supported by the International Vaccine Institute, Vaccine Impact Modelling Consortium (INV-034281) and the Japan Agency for Medical Research and Development (JP223fa627004).

## Data Statement

There were no original data collected nor data analyses generated for this review article.

## Declaration of Competing Interest

The authors declare the following financial interests/personal relationships which may be considered as potential competing interests: Following completion of the drafting of this document, David Beasley assumed responsibility for a Research Testing Agreement at the University of Texas Medical Branch funded by Valneva. SE related to evaluation of Chikungunya vaccine. All other authors declare no conflicts of interest.

## Data Availability

No data was used for the research described in the article.
