# Supplementary material for: Vaccine value profile for Chikungunya
Source: Vaccine. 2024 Jul 25;42(19 Suppl 1):S9–S24. doi: 10.1016/j.vaccine.2023.07.069 (PMC11554007; doi:10.1016/j.vaccine.2023.07.069)
Supplement: Supplementary data 1 [file mmc1.docx]

**Appendix A: Framework to inform section 5 - Vaccine development**

|  |  |  | | | | |
| --- | --- | --- | --- | --- | --- | --- |
| **Themes Considered** | **Indicators** | **Very Low** | **Low** | **Moderate** | **High** | **Very High** |
| **Biological Feasibility** | Most advanced vaccine candidate(s) | • Ph1, or preclinical, or no candidates in the pipeline | • Ph2 candidate | • Ph3 candidate | • Ph3 candidate with high likelihood of licensure by a WHO-listed national regulatory authority | • Licensed vaccine by a WHO-listed national regulatory authority |
| **Biological Feasibility** | Existence of immunity from natural exposure | • No evidence that natural exposure confers immunity | • Conflicting or minimum evidence | • Some evidence and/or immunity of limited duration | • Good evidence of relatively long-lasting immunity | • Well established that  natural exposure confers protects against severe disease  (or indicated vaccine outcome) with  durable immunity |
| **Biological Feasibility** | Understanding mechanisms of immunity | • Mechanisms of pathogen induced immunity unknown | • Very limited or conflicted understanding of pathogen-induced immunity and/or immune-enhanced disease | • Some understanding of pathogen- induced immunity and whether immune-enhanced disease exists | • Good understanding of pathogen- induced immunity, however some mechanisms remain unclear; evidence that immune-enhanced disease is unlikely | • All mechanisms of  pathogen-induced  immunity are well  understood; robust evidence that immune-enhanced disease is rare |
| **Biological Feasibility** | Likelihood of vaccine protection against the majority of pathogenic strains | • Evidence that a vaccine would not protect against majority of pathogenic strains, or gap in evidence of that a vaccine would protect against majority of pathogenic strains | • Limited evidence that a vaccine would protect against majority of pathogenic strains | • Some evidence that a vaccine would protect against majority of pathogenic strains | • Strong evidence that a vaccine would protect against majority of pathogenic strains | • No known strain  variation that is relevant  to a vaccine OR  evidence that a vaccine will protect against  all known strains OR new strain can be rapidly developed |
| **Product Development Feasibility** | Existence of animal models to facilitate vaccine development | • Animal models do not exist and no progress has been made to identify them. | • Animal models do not exist but some progress has been made to identify them. | • Animal models are identified but their utility have not been confirmed | • Animal models are identified and used but the mechanisms of immunity are unclear | • Animal models are well -defined and used and m  mechanisms of immunity are  well understood OR  animal models are not  required for vaccine  development |
| **Product Development Feasibility** | Existence of in vitro assays to facilitate vaccine development | • In vitro assays do not exist and no progress has been made to develop them. | • In vitro assays do not exist but some progress has been made to develop them. | • In vitro assays are developed but their utility has not been established or the assays have not been analytically qualified | • In vitro assays are analytically qualified and used but their fit for purpose as a relevant biomarker for decision-making or licensure has not been established | • In vitro assays are  qualified/validate  and are fit-for-purpose for decision-making or licensure |
| **Product Development Feasibility** | Ease of Clinical Development | • A complex trial design (no correlate) and a significant investment in clinical sites/infrastructure needed for testing a vaccine OR low disease incidence a significant impediment to efficacy trial feasibility | • A complex trial design needed (no correlate) that may require investment in clinical sites/infrastructure OR low disease incidence requires a large and/or long efficacy trial | • A standard trial design that may require investment in clinical sites/infrastructure | • Common trial design, potential correlate and a possibility to  leverage existing trial  infrastructure. | • Common trial design, established correlate  and a possibility to  leverage existing trial  infrastructure AND high disease  incidence allows for an  efficacy trial. |
| **Product Development Feasibility** | Availability or need for human challenge models | • Human challenge models OR diagnostic tools are important for vaccine development but are not developed. | • Human challenge models OR diagnostic tools are important for vaccine development and some progress has been made to develop them. | • Human challenge models OR diagnostic tools are important for vaccine development, are developed but not used or their use is unclear. | • Human challenge models OR diagnostic tools would facilitate vaccine development and are developed but their use is limited. | • Human challenge models  OR diagnostic tools are  important for vaccine  development, are developed  and widely used and accepted, OR no  human challenge model is  required |
