# Supplementary material for: Vaccine value profile for Chikungunya
Source: Vaccine. 2024 Jul 25;42(19 Suppl 1):S9–S24. doi: 10.1016/j.vaccine.2023.07.069 (PMC11554007; doi:10.1016/j.vaccine.2023.07.069)
Supplement: Supplementary data 2 [file mmc2.docx]

**Appendix B:** **Framework to inform section 8 - Access and Implementation Feasibility**

| **Themes Considered** | **Indicators** | **Very Low** | **Low** | **Moderate** | **High** | **Very High** |  |
| --- | --- | --- | --- | --- | --- | --- | --- |
| **Access and Implementation Feasibility** | Possibility of implementation within existing delivery systems | • No possibility to leverage existing delivery systems due to a complex vaccine immunisation schedule. | • Some evidence that existing delivery systems could be leveraged to deliver a vaccine | • Limited use of existing delivery systems to deliver a vaccine | • Vaccine can be delivered within existing delivery systems with amendments | • Vaccine can be delivered within existing delivery systems as is |  |
| **Access and Implementation Feasibility** | Commercial attractiveness | • Poorly defined target population • Disease burden mainly in LMICs but vaccine unlikely to be supported by Gavi | • Small target population predominantly in LMIC public markets • Difficulty defining target population in LMICs | • Large target population distributed predominantly in LMICs with potential Gavi support | • Well-defined target population in LMIC public markets • Large target populations distributed across HIC and LMIC markets | • Large target population in HIC and LMIC, both private and public markets |  |
| **Access and Implementation Feasibility** | Clarity of licensure and policy decision pathway | • A need for novel licensure and/or policy pathway, which is currently unclear | • A need for novel licensure and/or policy pathway | • A possibility to leverage an existing licensure and policy pathway with major amendments | • A clear licensure and policy pathway with minor amendments | • A clear, highly precedented, fit for purpose licensure and policy pathway currently exists |  |
| **Access and Implementation Feasibility** | Expected financing mechanism | No interest from global funders or national procurement agencies, potential for private market | Unlikely to be of interest to global funders, requiring commitment from national procurement | Potential interest from global funders, depending on public health impact data, interest from national procurement agencies | High level of interest expressed from public financing agencies such as Gavi, PAHO RF, and from national procurement agencies | Advanced purchasing commitment from, for example Gavi, PAHO RF, or other pull mechanism(s) in place |  |
| **Access and Implementation Feasibility** | Ease of uptake | • Extensive challenges with a new vaccination touchpoint required • High level of clinician judgement and clinical engagement • Additional extensive barriers to uptake including lack of national commitment | • Evidence of low uptake for marketed vaccines • Cultural barriers, negative patient perceptions | • New vaccination touchpoint required | •Well-defined target population with likelihood of high acceptability, but possible difficulties in infrastructure for vaccination | •Well-defined target population with likelihood of high acceptability • Evidence of high uptake for marketed vaccine • Lack of other significant barriers to introduce a vaccine • Strong national commitment to introduce a vaccine |  |
